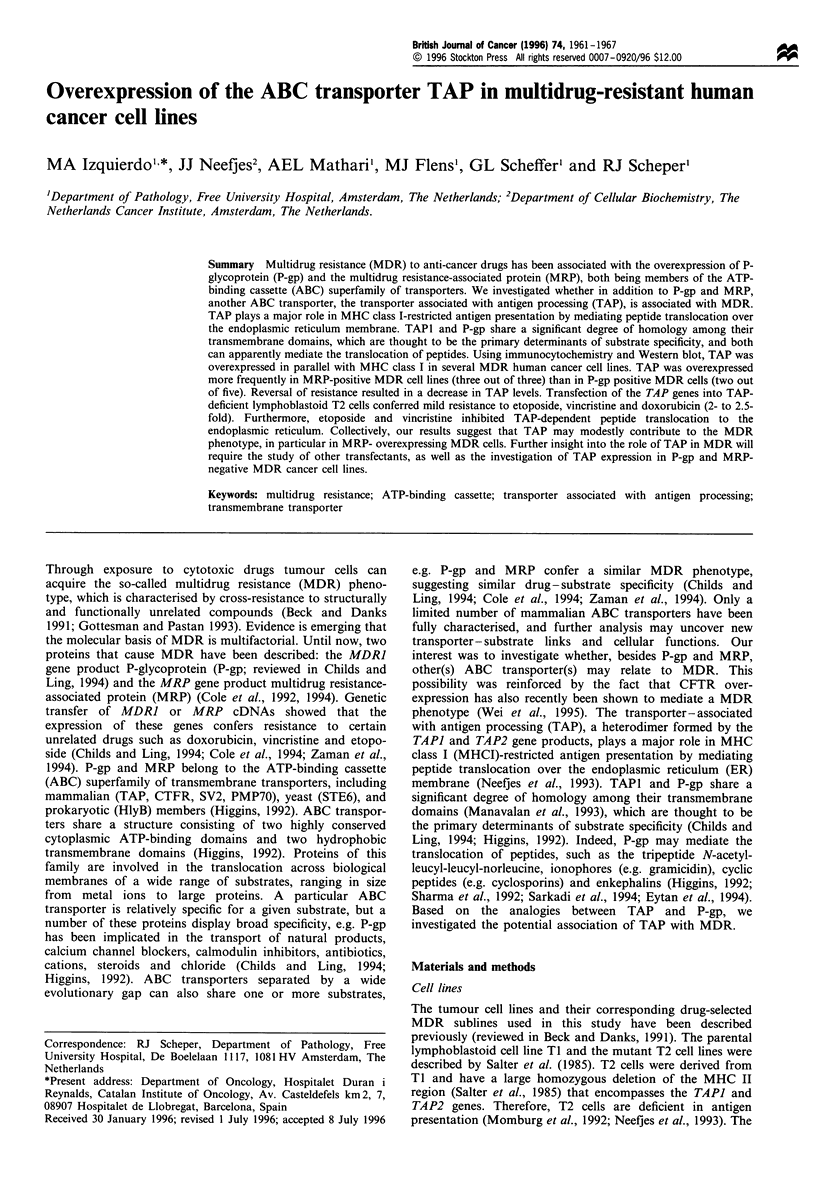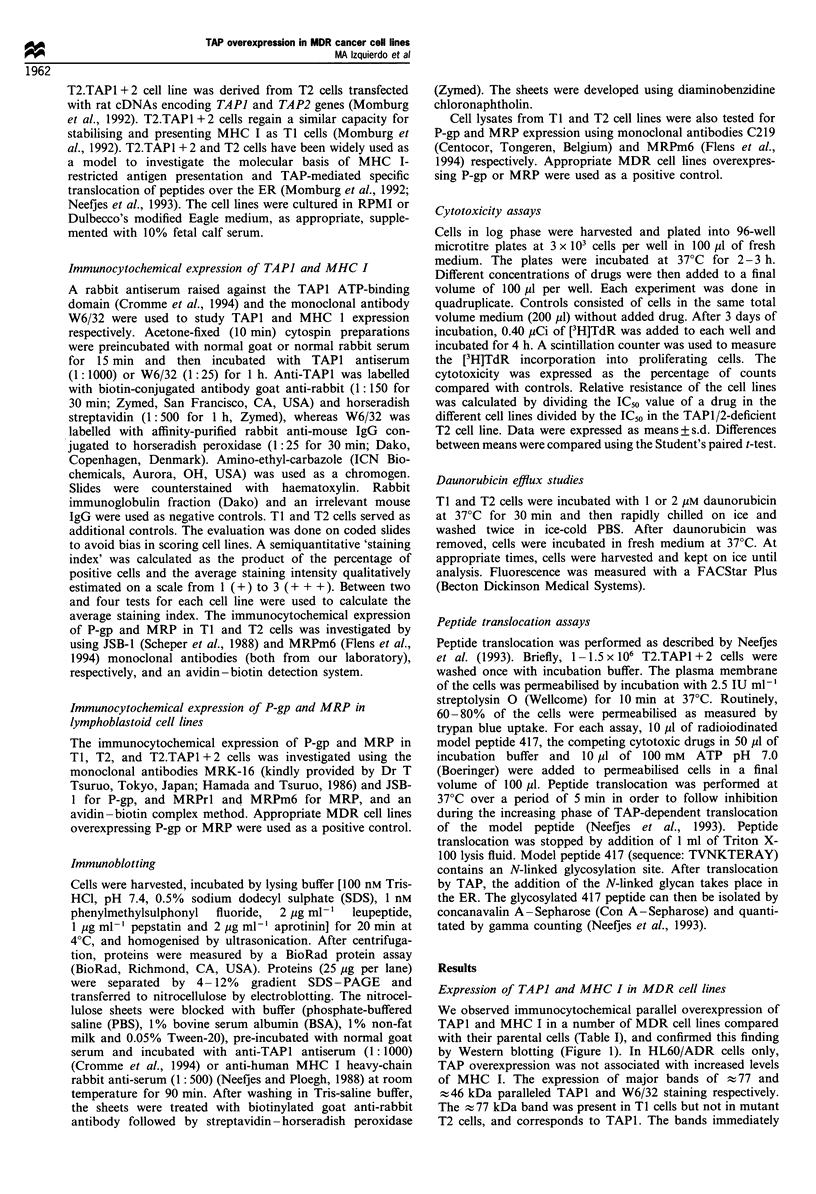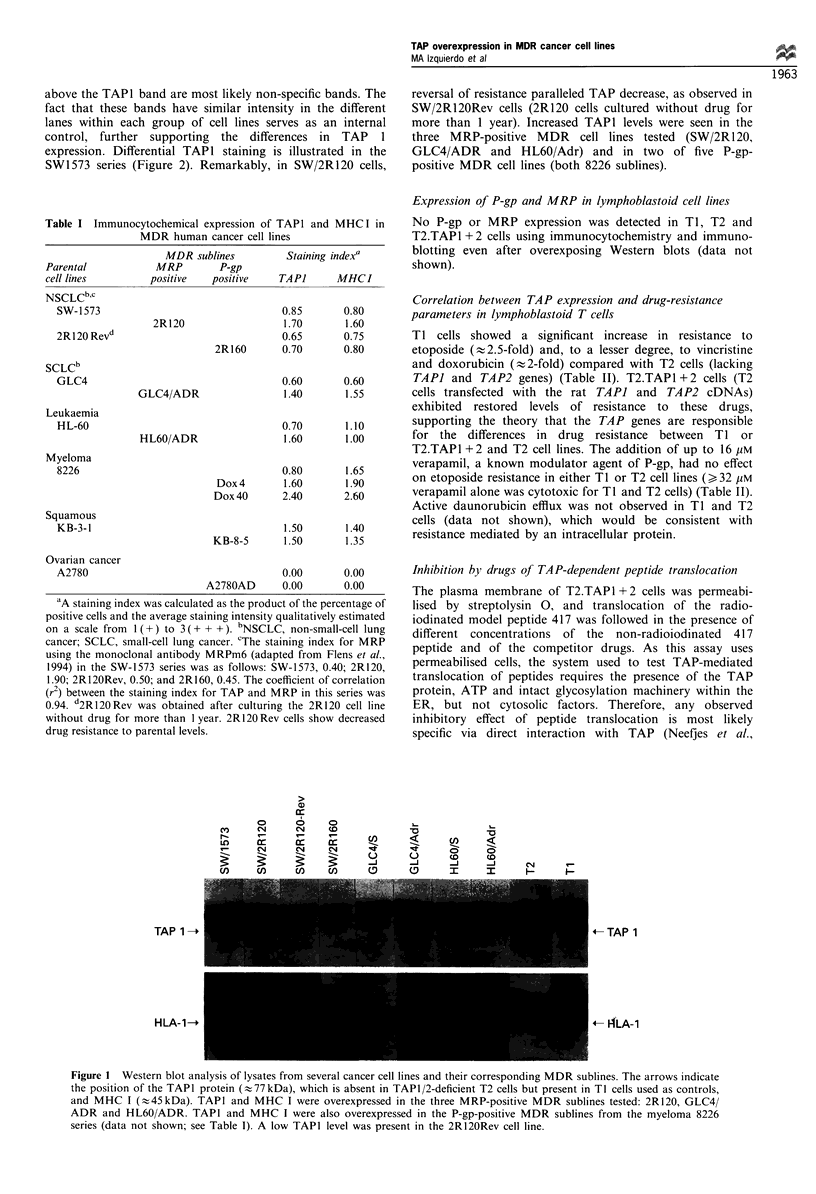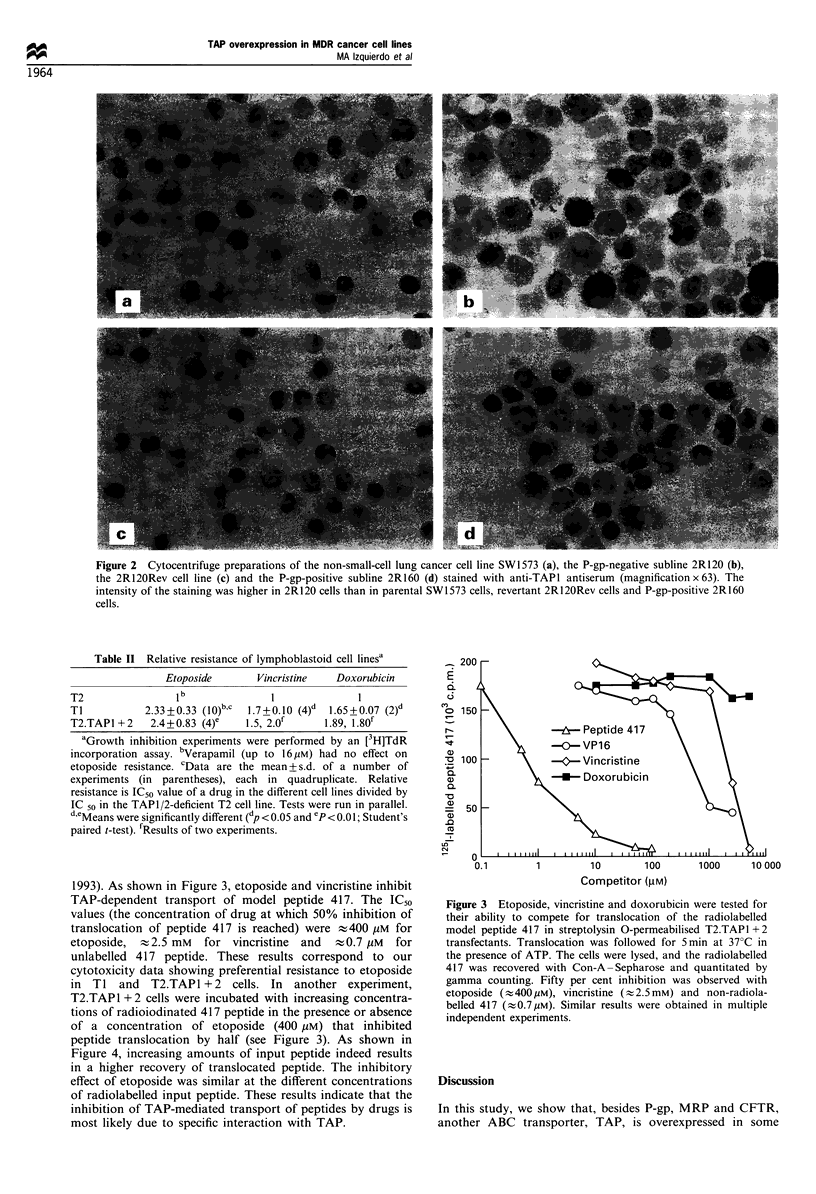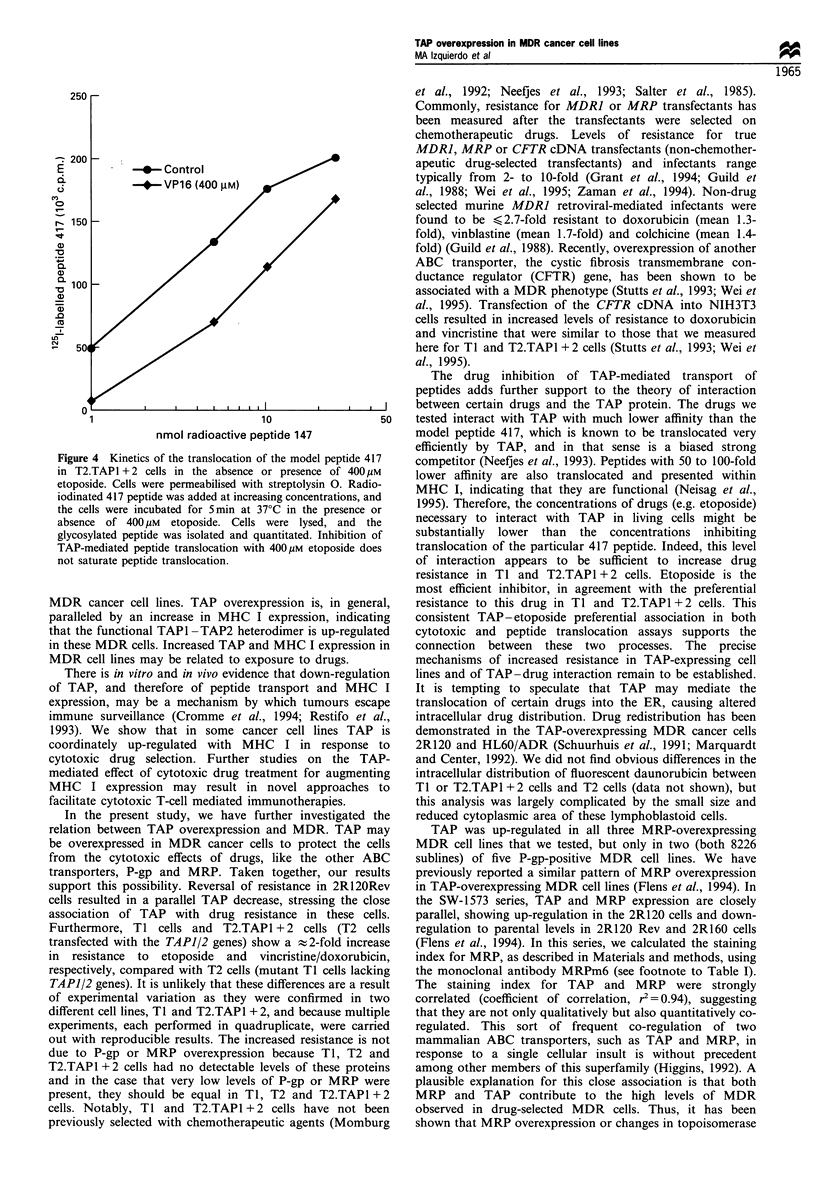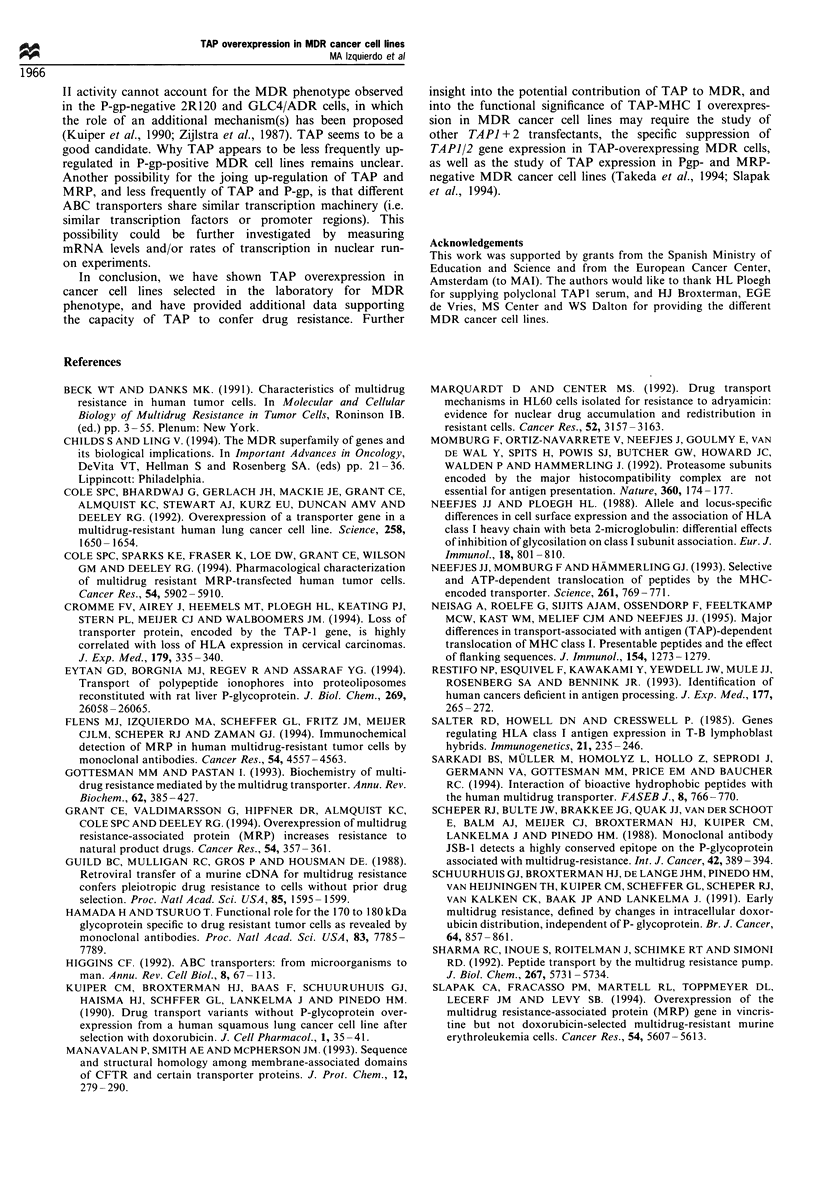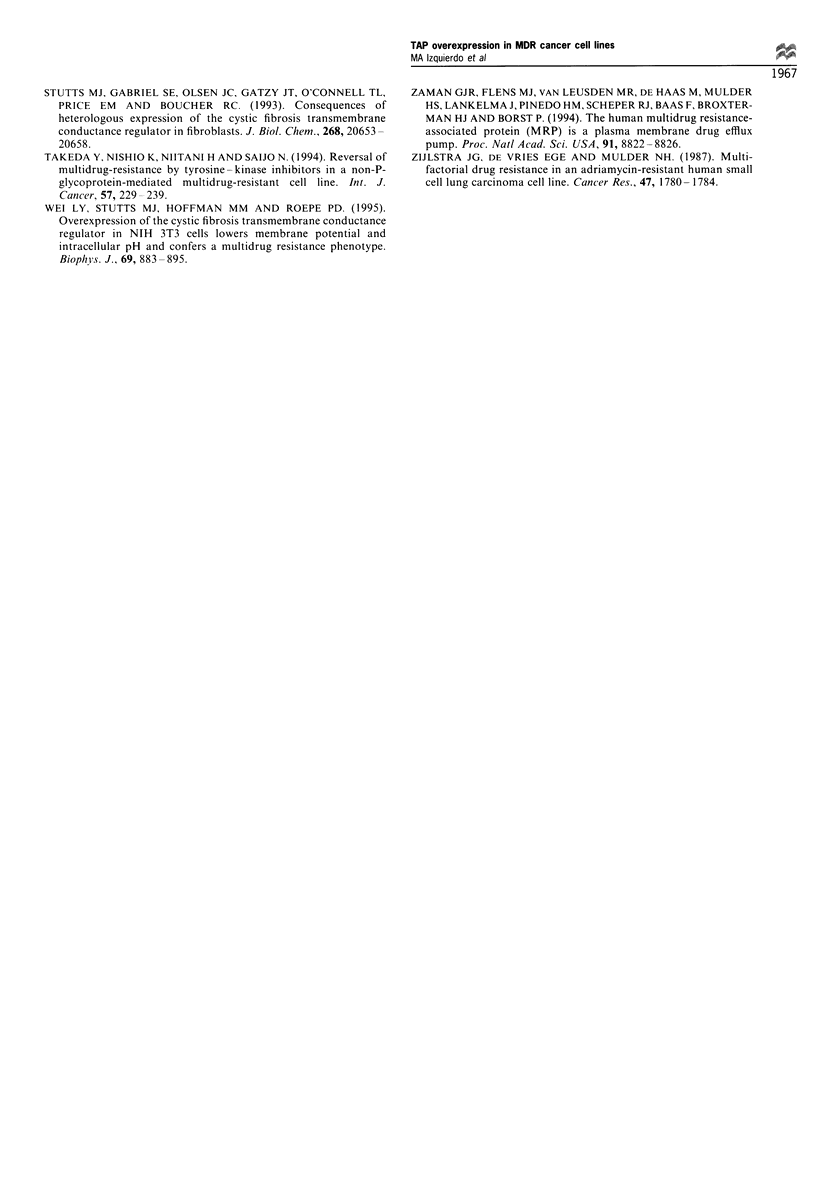# Overexpression of the ABC transporter TAP in multidrug-resistant human cancer cell lines.

**DOI:** 10.1038/bjc.1996.660

**Published:** 1996-12

**Authors:** M. A. Izquierdo, J. J. Neefjes, A. E. Mathari, M. J. Flens, G. L. Scheffer, R. J. Scheper

**Affiliations:** Department of Pathology, Free University Hospital, Amsterdam, The Netherlands.

## Abstract

**Images:**


British Journal of Cancer (1996) 74, 1961-1967
© 1996 Stockton Press All rights reserved 0007-0920/96 $12.00

### Overexpression of the ABC transporter TAP in multidrug-resistant human cancer cell lines

MA Izquierdo[1,*], JJ Neefjes[2], AEL Mathari[1], MJ Flens[1], GL Scheffer[1] and RJ Scheper[1]

[1]Department of Pathology, Free University Hospital, Amsterdam, The Netherlands; [2]Department of Cellular Biochemistry, The Netherlands Cancer Institute, Amsterdam, The Netherlands.

**Summary** Multidrug resistance (MDR) to anti-cancer drugs has been associated with the overexpression of P-glycoprotein (P-gp) and the multidrug resistance-associated protein (MRP), both being members of the ATP-binding cassette (ABC) superfamily of transporters. We investigated whether in addition to P-gp and MRP, another ABC transporter, the transporter associated with antigen processing (TAP), is associated with MDR. TAP plays a major role in MHC class I-restricted antigen presentation by mediating peptide translocation over the endoplasmic reticulum membrane. TAP1 and P-gp share a significant degree of homology among their transmembrane domains, which are thought to be the primary determinants of substrate specificity, and both can apparently mediate the translocation of peptides. Using immunocytochemistry and Western blot, TAP was overexpressed in parallel with MHC class I in several MDR human cancer cell lines. TAP was overexpressed more frequently in MRP-positive MDR cell lines (three out of three) than in P-gp positive MDR cells (two out of five). Reversal of resistance resulted in a decrease in TAP levels. Transfection of the *TAP* genes into TAP-deficient lymphoblastoid T2 cells conferred mild resistance to etoposide, vincristine and doxorubicin (2- to 2.5-fold). Furthermore, etoposide and vincristine inhibited TAP-dependent peptide translocation to the endoplasmic reticulum. Collectively, our results suggest that TAP may modestly contribute to the MDR phenotype, in particular in MRP- overexpressing MDR cells. Further insight into the role of TAP in MDR will require the study of other transfectants, as well as the investigation of TAP expression in P-gp and MRP-negative MDR cancer cell lines.

**Keywords:** multidrug resistance; ATP-binding cassette; transporter associated with antigen processing; transmembrane transporter

Through exposure to cytotoxic drugs tumour cells can acquire the so-called multidrug resistance (MDR) phenotype, which is characterised by cross-resistance to structurally and functionally unrelated compounds (Beck and Danks 1991; Gottesman and Pastan 1993). Evidence is emerging that the molecular basis of MDR is multifactorial. Until now, two proteins that cause MDR have been described: the *MDR1* gene product P-glycoprotein (P-gp; reviewed in Childs and Ling, 1994) and the *MRP* gene product multidrug resistance-associated protein (MRP) (Cole *et al.*, 1992, 1994). Genetic transfer of *MDR1* or *MRP* cDNAs showed that the expression of these genes confers resistance to certain unrelated drugs such as doxorubicin, vincristine and etoposide (Childs and Ling, 1994; Cole *et al.*, 1994; Zaman *et al.*, 1994). P-gp and MRP belong to the ATP-binding cassette (ABC) superfamily of transmembrane transporters, including mammalian (TAP, CTFR, SV2, PMP70), yeast (STE6), and prokaryotic (HlyB) members (Higgins, 1992). ABC transporters share a structure consisting of two highly conserved cytoplasmic ATP-binding domains and two hydrophobic transmembrane domains (Higgins, 1992). Proteins of this family are involved in the translocation across biological membranes of a wide range of substrates, ranging in size from metal ions to large proteins. A particular ABC transporter is relatively specific for a given substrate, but a number of these proteins display broad specificity, e.g. P-gp has been implicated in the transport of natural products, calcium channel blockers, calmodulin inhibitors, antibiotics, cations, steroids and chloride (Childs and Ling, 1994; Higgins, 1992). ABC transporters separated by a wide evolutionary gap can also share one or more substrates, e.g. P-gp and MRP confer a similar MDR phenotype, suggesting similar drug–substrate specificity (Childs and Ling, 1994; Cole *et al.*, 1994; Zaman *et al.*, 1994). Only a limited number of mammalian ABC transporters have been fully characterised, and further analysis may uncover new transporter–substrate links and cellular functions. Our interest was to investigate whether, besides P-gp and MRP, other(s) ABC transporter(s) may relate to MDR. This possibility was reinforced by the fact that CFTR overexpression has also recently been shown to mediate a MDR phenotype (Wei *et al.*, 1995). The transporter–associated with antigen processing (TAP), a heterodimer formed by the *TAP1* and *TAP2* gene products, plays a major role in MHC class I (MHCI)-restricted antigen presentation by mediating peptide translocation over the endoplasmic reticulum (ER) membrane (Neefjes *et al.*, 1993). TAP1 and P-gp share a significant degree of homology among their transmembrane domains (Manavalan *et al.*, 1993), which are thought to be the primary determinants of substrate specificity (Childs and Ling, 1994; Higgins, 1992). Indeed, P-gp may mediate the translocation of peptides, such as the tripeptide *N*-acetyl-leucyl-leucyl-norleucine, ionophores (e.g. gramicidin), cyclic peptides (e.g. cyclosporins) and enkephalins (Higgins, 1992; Sharma *et al.*, 1992; Sarkadi *et al.*, 1994; Eytan *et al.*, 1994). Based on the analogies between TAP and P-gp, we investigated the potential association of TAP with MDR.

### Materials and methods

### Cell lines

The tumour cell lines and their corresponding drug-selected MDR sublines used in this study have been described previously (reviewed in Beck and Danks, 1991). The parental lymphoblastoid cell line T1 and the mutant T2 cell lines were described by Salter *et al.* (1985). T2 cells were derived from T1 and have a large homozygous deletion of the MHC II region (Salter *et al.*, 1985) that encompasses the *TAP1* and *TAP2* genes. Therefore, T2 cells are deficient in antigen presentation (Momburg *et al.*, 1992; Neefjes *et al.*, 1993). The

Correspondence: RJ Scheper, Department of Pathology, Free University Hospital, De Boelelaan 1117, 1081HV Amsterdam, The Netherlands
*Present address: Department of Oncology, Hospitalet Duran i Reynalds, Catalan Institute of Oncology, Av. Casteldefels km 2, 7, 08907 Hospitalet de Llobregat, Barcelona, Spain
Received 30 January 1996; revised 1 July 1996; accepted 8 July 1996

T2.TAP1+2 cell line was derived from T2 cells transfected with rat cDNAs encoding *TAP1* and *TAP2* genes (Momburg *et al.*, 1992). T2.TAP1+2 cells regain a similar capacity for stabilising and presenting MHC I as T1 cells (Momburg *et al.*, 1992). T2.TAP1+2 and T2 cells have been widely used as a model to investigate the molecular basis of MHC I-restricted antigen presentation and TAP-mediated specific translocation of peptides over the ER (Momburg *et al.*, 1992; Neefjes *et al.*, 1993). The cell lines were cultured in RPMI or Dulbecco's modified Eagle medium, as appropriate, supplemented with 10% fetal calf serum.

### *Immunocytochemical expression of TAP1 and MHC I*

A rabbit antiserum raised against the TAP1 ATP-binding domain (Cromme *et al.*, 1994) and the monoclonal antibody W6/32 were used to study TAP1 and MHC 1 expression respectively. Acetone-fixed (10 min) cytospin preparations were preincubated with normal goat or normal rabbit serum for 15 min and then incubated with TAP1 antiserum (1:1000) or W6/32 (1:25) for 1 h. Anti-TAP1 was labelled with biotin-conjugated antibody goat anti-rabbit (1:150 for 30 min; Zymed, San Francisco, CA, USA) and horseradish streptavidin (1:500 for 1 h, Zymed), whereas W6/32 was labelled with affinity-purified rabbit anti-mouse IgG conjugated to horseradish peroxidase (1:25 for 30 min; Dako, Copenhagen, Denmark). Amino-ethyl-carbazole (ICN Biochemicals, Aurora, OH, USA) was used as a chromogen. Slides were counterstained with haematoxylin. Rabbit immunoglobulin fraction (Dako) and an irrelevant mouse IgG were used as negative controls. T1 and T2 cells served as additional controls. The evaluation was done on coded slides to avoid bias in scoring cell lines. A semiquantitative 'staining index' was calculated as the product of the percentage of positive cells and the average staining intensity qualitatively estimated on a scale from 1 (+) to 3 (+ + +). Between two and four tests for each cell line were used to calculate the average staining index. The immunocytochemical expression of P-gp and MRP in T1 and T2 cells was investigated by using JSB-1 (Scheper *et al.*, 1988) and MRPm6 (Flens *et al.*, 1994) monoclonal antibodies (both from our laboratory), respectively, and an avidin–biotin detection system.

### *Immunocytochemical expression of P-gp and MRP in lymphoblastoid cell lines*

The immunocytochemical expression of P-gp and MRP in T1, T2, and T2.TAP1+2 cells was investigated using the monoclonal antibodies MRK-16 (kindly provided by Dr T Tsuruo, Tokyo, Japan; Hamada and Tsuruo, 1986) and JSB-1 for P-gp, and MRPr1 and MRPm6 for MRP, and an avidin–biotin complex method. Appropriate MDR cell lines overexpressing P-gp or MRP were used as a positive control.

### *Immunoblotting*

Cells were harvested, incubated by lysing buffer [100 nM Tris-HCl, pH 7.4, 0.5% sodium dodecyl sulphate (SDS), 1 nM phenylmethylsulphonyl fluoride, 2 $\mu$g ml$^{-1}$ leupeptide, 1 $\mu$g ml$^{-1}$ pepstatin and 2 $\mu$g ml$^{-1}$ aprotinin] for 20 min at 4°C, and homogenised by ultrasonication. After centrifugation, proteins were measured by a BioRad protein assay (BioRad, Richmond, CA, USA). Proteins (25 $\mu$g per lane) were separated by 4–12% gradient SDS–PAGE and transferred to nitrocellulose by electroblotting. The nitrocellulose sheets were blocked with buffer (phosphate-buffered saline (PBS), 1% bovine serum albumin (BSA), 1% non-fat milk and 0.05% Tween-20), pre-incubated with normal goat serum and incubated with anti-TAP1 antiserum (1:1000) (Cromme *et al.*, 1994) or anti-human MHC I heavy-chain rabbit anti-serum (1:500) (Neefjes and Ploegh, 1988) at room temperature for 90 min. After washing in Tris-saline buffer, the sheets were treated with biotinylated goat anti-rabbit antibody followed by streptavidin–horseradish peroxidase (Zymed). The sheets were developed using diaminobenzidine chloronaphtholin.

Cell lysates from T1 and T2 cell lines were also tested for P-gp and MRP expression using monoclonal antibodies C219 (Centocor, Tongeren, Belgium) and MRPm6 (Flens *et al.*, 1994) respectively. Appropriate MDR cell lines overexpressing P-gp or MRP were used as a positive control.

### *Cytotoxicity assays*

Cells in log phase were harvested and plated into 96-well microtitre plates at $3 \times 10^3$ cells per well in 100 $\mu$l of fresh medium. The plates were incubated at 37°C for 2–3 h. Different concentrations of drugs were then added to a final volume of 100 $\mu$l per well. Each experiment was done in quadruplicate. Controls consisted of cells in the same total volume medium (200 $\mu$l) without added drug. After 3 days of incubation, 0.40 $\mu$Ci of [$^3$H]TdR was added to each well and incubated for 4 h. A scintillation counter was used to measure the [$^3$H]TdR incorporation into proliferating cells. The cytotoxicity was expressed as the percentage of counts compared with controls. Relative resistance of the cell lines was calculated by dividing the $IC_{50}$ value of a drug in the different cell lines divided by the $IC_{50}$ in the TAP1/2-deficient T2 cell line. Data were expressed as means ± s.d. Differences between means were compared using the Student's paired *t*-test.

### *Daunorubicin efflux studies*

T1 and T2 cells were incubated with 1 or 2 $\mu$M daunorubicin at 37°C for 30 min and then rapidly chilled on ice and washed twice in ice-cold PBS. After daunorubicin was removed, cells were incubated in fresh medium at 37°C. At appropriate times, cells were harvested and kept on ice until analysis. Fluorescence was measured with a FACStar Plus (Becton Dickinson Medical Systems).

### *Peptide translocation assays*

Peptide translocation was performed as described by Neefjes *et al.* (1993). Briefly, $1-1.5 \times 10^6$ T2.TAP1+2 cells were washed once with incubation buffer. The plasma membrane of the cells was permeabilised by incubation with 2.5 IU ml$^{-1}$ streptolysin O (Wellcome) for 10 min at 37°C. Routinely, 60–80% of the cells were permeabilised as measured by trypan blue uptake. For each assay, 10 $\mu$l of radioiodinated model peptide 417, the competing cytotoxic drugs in 50 $\mu$l of incubation buffer and 10 $\mu$l of 100 mM ATP pH 7.0 (Boeringer) were added to permeabilised cells in a final volume of 100 $\mu$l. Peptide translocation was performed at 37°C over a period of 5 min in order to follow inhibition during the increasing phase of TAP-dependent translocation of the model peptide (Neefjes *et al.*, 1993). Peptide translocation was stopped by addition of 1 ml of Triton X-100 lysis fluid. Model peptide 417 (sequence: TVNKTERAY) contains an *N*-linked glycosylation site. After translocation by TAP, the addition of the *N*-linked glycan takes place in the ER. The glycosylated 417 peptide can then be isolated by concanavalin A–Sepharose (Con A–Sepharose) and quantitated by gamma counting (Neefjes *et al.*, 1993).

### Results

### *Expression of TAP1 and MHC I in MDR cell lines*

We observed immunocytochemical parallel overexpression of TAP1 and MHC I in a number of MDR cell lines compared with their parental cells (Table I), and confirmed this finding by Western blotting (Figure 1). In HL60/ADR cells only, TAP overexpression was not associated with increased levels of MHC I. The expression of major bands of ≈77 and ≈46 kDa paralleled TAP1 and W6/32 staining respectively. The ≈77 kDa band was present in T1 cells but not in mutant T2 cells, and corresponds to TAP1. The bands immediately above the TAP1 band are most likely non-specific bands. The fact that these bands have similar intensity in the different lanes within each group of cell lines serves as an internal control, further supporting the differences in TAP 1 expression. Differential TAP1 staining is illustrated in the SW1573 series (Figure 2). Remarkably, in SW/2R120 cells, reversal of resistance paralleled TAP decrease, as observed in SW/2R120Rev cells (2R120 cells cultured without drug for more than 1 year). Increased TAP1 levels were seen in the three MRP-positive MDR cell lines tested (SW/2R120, GLC4/ADR and HL60/Adr) and in two of five P-gp-positive MDR cell lines (both 8226 sublines).

**Table I** Immunocytochemical expression of TAP1 and MHC I in MDR human cancer cell lines

| *Parental cell lines* | *MDR sublines* *MRP positive* | *P-gp positive* | *Staining index*[a] *TAP1* | *MHC I* |
|---|---|---|---|---|
| NSCLC[b,c] | | | | |
| SW-1573 | | | 0.85 | 0.80 |
| | 2R120 | | 1.70 | 1.60 |
| 2R120 Rev[d] | | | 0.65 | 0.75 |
| | | 2R160 | 0.70 | 0.80 |
| SCLC[b] | | | | |
| GLC4 | | | 0.60 | 0.60 |
| | GLC4/ADR | | 1.40 | 1.55 |
| Leukaemia | | | | |
| HL-60 | | | 0.70 | 1.10 |
| | HL60/ADR | | 1.60 | 1.00 |
| Myeloma | | | | |
| 8226 | | | 0.80 | 1.65 |
| | | Dox 4 | 1.60 | 1.90 |
| | | Dox 40 | 2.40 | 2.60 |
| Squamous | | | | |
| KB-3-1 | | | 1.50 | 1.40 |
| | | KB-8-5 | 1.50 | 1.35 |
| Ovarian cancer | | | | |
| A2780 | | | 0.00 | 0.00 |
| | | A2780AD | 0.00 | 0.00 |

[a]A staining index was calculated as the product of the percentage of positive cells and the average staining intensity qualitatively estimated on a scale from 1(+) to 3(+ + +). [b]NSCLC, non-small-cell lung cancer; SCLC, small-cell lung cancer. [c]The staining index for MRP using the monoclonal antibody MRPm6 (adapted from Flens *et al.*, 1994) in the SW-1573 series was as follows: SW-1573, 0.40; 2R120, 1.90; 2R120Rev, 0.50; and 2R160, 0.45. The coefficient of correlation ($r^2$) between the staining index for TAP and MRP in this series was 0.94. [d]2R120 Rev was obtained after culturing the 2R120 cell line without drug for more than 1 year. 2R120 Rev cells show decreased drug resistance to parental levels.

*Expression of P-gp and MRP in lymphoblastoid cell lines*

No P-gp or MRP expression was detected in T1, T2 and T2.TAP1 + 2 cells using immunocytochemistry and immunoblotting even after overexposing Western blots (data not shown).

*Correlation between TAP expression and drug-resistance parameters in lymphoblastoid T cells*

T1 cells showed a significant increase in resistance to etoposide (≈2.5-fold) and, to a lesser degree, to vincristine and doxorubicin (≈2-fold) compared with T2 cells (lacking *TAP1* and *TAP2* genes) (Table II). T2.TAP1 + 2 cells (T2 cells transfected with the rat *TAP1* and *TAP2* cDNAs) exhibited restored levels of resistance to these drugs, supporting the theory that the *TAP* genes are responsible for the differences in drug resistance between T1 or T2.TAP1 + 2 and T2 cell lines. The addition of up to 16 $\mu$M verapamil, a known modulator agent of P-gp, had no effect on etoposide resistance in either T1 or T2 cell lines (≥32 $\mu$M verapamil alone was cytotoxic for T1 and T2 cells) (Table II). Active daunorubicin efflux was not observed in T1 and T2 cells (data not shown), which would be consistent with resistance mediated by an intracellular protein.

*Inhibition by drugs of TAP-dependent peptide translocation*

The plasma membrane of T2.TAP1 + 2 cells was permeabilised by streptolysin O, and translocation of the radioiodinated model peptide 417 was followed in the presence of different concentrations of the non-radioiodinated 417 peptide and of the competitor drugs. As this assay uses permeabilised cells, the system used to test TAP-mediated translocation of peptides requires the presence of the TAP protein, ATP and intact glycosylation machinery within the ER, but not cytosolic factors. Therefore, any observed inhibitory effect of peptide translocation is most likely specific via direct interaction with TAP (Neefjes *et al.*,

**Figure 1** Western blot analysis of lysates from several cancer cell lines and their corresponding MDR sublines. The arrows indicate the position of the TAP1 protein (≈77 kDa), which is absent in TAP1/2-deficient T2 cells but present in T1 cells used as controls, and MHC I (≈45 kDa). TAP1 and MHC I were overexpressed in the three MRP-positive MDR sublines tested: 2R120, GLC4/ADR and HL60/ADR. TAP1 and MHC I were also overexpressed in the P-gp-positive MDR sublines from the myeloma 8226 series (data not shown; see Table I). A low TAP1 level was present in the 2R120Rev cell line.

**Figure 2** Cytocentrifuge preparations of the non-small-cell lung cancer cell line SW1573 (**a**), the P-gp-negative subline 2R120 (**b**), the 2R120Rev cell line (**c**) and the P-gp-positive subline 2R160 (**d**) stained with anti-TAP1 antiserum (magnification × 63). The intensity of the staining was higher in 2R120 cells than in parental SW1573 cells, revertant 2R120Rev cells and P-gp-positive 2R160 cells.

**Table II** Relative resistance of lymphoblastoid cell lines[a]

| | *Etoposide* | *Vincristine* | *Doxorubicin* |
|---|---|---|---|
| T2 | 1[b] | 1 | 1 |
| T1 | 2.33±0.33 (10)[b,c] | 1.7±0.10 (4)[d] | 1.65±0.07 (2)[d] |
| T2.TAP1+2 | 2.4±0.83 (4)[e] | 1.5, 2.0[f] | 1.89, 1.80[f] |

[a]Growth inhibition experiments were performed by an [$^3$H]TdR incorporation assay. [b]Verapamil (up to 16 $\mu$M) had no effect on etoposide resistance. [c]Data are the mean ± s.d. of a number of experiments (in parentheses), each in quadruplicate. Relative resistance is $IC_{50}$ value of a drug in the different cell lines divided by $IC_{50}$ in the TAP1/2-deficient T2 cell line. Tests were run in parallel. [d,e]Means were significantly different ([d]$p<0.05$ and [e]$P<0.01$; Student's paired $t$-test). [f]Results of two experiments.

1993). As shown in Figure 3, etoposide and vincristine inhibit TAP-dependent transport of model peptide 417. The $IC_{50}$ values (the concentration of drug at which 50% inhibition of translocation of peptide 417 is reached) were ≈400 $\mu$M for etoposide, ≈2.5 mM for vincristine and ≈0.7 $\mu$M for unlabelled 417 peptide. These results correspond to our cytotoxicity data showing preferential resistance to etoposide in T1 and T2.TAP1+2 cells. In another experiment, T2.TAP1+2 cells were incubated with increasing concentrations of radioiodinated 417 peptide in the presence or absence of a concentration of etoposide (400 $\mu$M) that inhibited peptide translocation by half (see Figure 3). As shown in Figure 4, increasing amounts of input peptide indeed results in a higher recovery of translocated peptide. The inhibitory effect of etoposide was similar at the different concentrations of radiolabelled input peptide. These results indicate that the inhibition of TAP-mediated transport of peptides by drugs is most likely due to specific interaction with TAP.

**Figure 3** Etoposide, vincristine and doxorubicin were tested for their ability to compete for translocation of the radiolabelled model peptide 417 in streptolysin O-permeabilised T2.TAP1+2 transfectants. Translocation was followed for 5 min at 37°C in the presence of ATP. The cells were lysed, and the radiolabelled 417 was recovered with Con-A–Sepharose and quantitated by gamma counting. Fifty per cent inhibition was observed with etoposide (≈400 $\mu$M), vincristine (≈2.5 mM) and non-radiolabelled 417 (≈0.7 $\mu$M). Similar results were obtained in multiple independent experiments.

### Discussion

In this study, we show that, besides P-gp, MRP and CFTR, another ABC transporter, TAP, is overexpressed in some

**Figure 4** Kinetics of the translocation of the model peptide 417 in T2.TAP1+2 cells in the absence or presence of 400 μM etoposide. Cells were permeabilised with streptolysin O. Radioiodinated 417 peptide was added at increasing concentrations, and the cells were incubated for 5 min at 37°C in the presence or absence of 400 μM etoposide. Cells were lysed, and the glycosylated peptide was isolated and quantitated. Inhibition of TAP-mediated peptide translocation with 400 μM etoposide does not saturate peptide translocation.

MDR cancer cell lines. TAP overexpression is, in general, paralleled by an increase in MHC I expression, indicating that the functional TAP1–TAP2 heterodimer is up-regulated in these MDR cells. Increased TAP and MHC I expression in MDR cell lines may be related to exposure to drugs.

There is *in vitro* and *in vivo* evidence that down-regulation of TAP, and therefore of peptide transport and MHC I expression, may be a mechanism by which tumours escape immune surveillance (Cromme *et al.*, 1994; Restifo *et al.*, 1993). We show that in some cancer cell lines TAP is coordinately up-regulated with MHC I in response to cytotoxic drug selection. Further studies on the TAP-mediated effect of cytotoxic drug treatment for augmenting MHC I expression may result in novel approaches to facilitate cytotoxic T-cell mediated immunotherapies.

In the present study, we have further investigated the relation between TAP overexpression and MDR. TAP may be overexpressed in MDR cancer cells to protect the cells from the cytotoxic effects of drugs, like the other ABC transporters, P-gp and MRP. Taken together, our results support this possibility. Reversal of resistance in 2R120Rev cells resulted in a parallel TAP decrease, stressing the close association of TAP with drug resistance in these cells. Furthermore, T1 cells and T2.TAP1+2 cells (T2 cells transfected with the *TAP1/2* genes) show a ≈2-fold increase in resistance to etoposide and vincristine/doxorubicin, respectively, compared with T2 cells (mutant T1 cells lacking *TAP1/2* genes). It is unlikely that these differences are a result of experimental variation as they were confirmed in two different cell lines, T1 and T2.TAP1+2, and because multiple experiments, each performed in quadruplicate, were carried out with reproducible results. The increased resistance is not due to P-gp or MRP overexpression because T1, T2 and T2.TAP1+2 cells had no detectable levels of these proteins and in the case that very low levels of P-gp or MRP were present, they should be equal in T1, T2 and T2.TAP1+2 cells. Notably, T1 and T2.TAP1+2 cells have not been previously selected with chemotherapeutic agents (Momburg *et al.*, 1992; Neefjes *et al.*, 1993; Salter *et al.*, 1985). Commonly, resistance for *MDR1* or *MRP* transfectants has been measured after the transfectants were selected on chemotherapeutic drugs. Levels of resistance for true *MDR1*, *MRP* or *CFTR* cDNA transfectants (non-chemotherapeutic drug-selected transfectants) and infectants range typically from 2- to 10-fold (Grant *et al.*, 1994; Guild *et al.*, 1988; Wei *et al.*, 1995; Zaman *et al.*, 1994). Non-drug selected murine *MDR1* retroviral-mediated infectants were found to be ⩽2.7-fold resistant to doxorubicin (mean 1.3-fold), vinblastine (mean 1.7-fold) and colchicine (mean 1.4-fold) (Guild *et al.*, 1988). Recently, overexpression of another ABC transporter, the cystic fibrosis transmembrane conductance regulator (CFTR) gene, has been shown to be associated with a MDR phenotype (Stutts *et al.*, 1993; Wei *et al.*, 1995). Transfection of the *CFTR* cDNA into NIH3T3 cells resulted in increased levels of resistance to doxorubicin and vincristine that were similar to those that we measured here for T1 and T2.TAP1+2 cells (Stutts *et al.*, 1993; Wei *et al.*, 1995).

The drug inhibition of TAP-mediated transport of peptides adds further support to the theory of interaction between certain drugs and the TAP protein. The drugs we tested interact with TAP with much lower affinity than the model peptide 417, which is known to be translocated very efficiently by TAP, and in that sense is a biased strong competitor (Neefjes *et al.*, 1993). Peptides with 50 to 100-fold lower affinity are also translocated and presented within MHC I, indicating that they are functional (Neisag *et al.*, 1995). Therefore, the concentrations of drugs (e.g. etoposide) necessary to interact with TAP in living cells might be substantially lower than the concentrations inhibiting translocation of the particular 417 peptide. Indeed, this level of interaction appears to be sufficient to increase drug resistance in T1 and T2.TAP1+2 cells. Etoposide is the most efficient inhibitor, in agreement with the preferential resistance to this drug in T1 and T2.TAP1+2 cells. This consistent TAP–etoposide preferential association in both cytotoxic and peptide translocation assays supports the connection between these two processes. The precise mechanisms of increased resistance in TAP-expressing cell lines and of TAP–drug interaction remain to be established. It is tempting to speculate that TAP may mediate the translocation of certain drugs into the ER, causing altered intracellular drug distribution. Drug redistribution has been demonstrated in the TAP-overexpressing MDR cancer cells 2R120 and HL60/ADR (Schuurhuis *et al.*, 1991; Marquardt and Center, 1992). We did not find obvious differences in the intracellular distribution of fluorescent daunorubicin between T1 or T2.TAP1+2 cells and T2 cells (data not shown), but this analysis was largely complicated by the small size and reduced cytoplasmic area of these lymphoblastoid cells.

TAP was up-regulated in all three MRP-overexpressing MDR cell lines that we tested, but only in two (both 8226 sublines) of five P-gp-positive MDR cell lines. We have previously reported a similar pattern of MRP overexpression in TAP-overexpressing MDR cell lines (Flens *et al.*, 1994). In the SW-1573 series, TAP and MRP expression are closely parallel, showing up-regulation in the 2R120 cells and down-regulation to parental levels in 2R120 Rev and 2R160 cells (Flens *et al.*, 1994). In this series, we calculated the staining index for MRP, as described in Materials and methods, using the monoclonal antibody MRPm6 (see footnote to Table I). The staining index for TAP and MRP were strongly correlated (coefficient of correlation, $r^2 = 0.94$), suggesting that they are not only qualitatively but also quantitatively co-regulated. This sort of frequent co-regulation of two mammalian ABC transporters, such as TAP and MRP, in response to a single cellular insult is without precedent among other members of this superfamily (Higgins, 1992). A plausible explanation for this close association is that both MRP and TAP contribute to the high levels of MDR observed in drug-selected MDR cells. Thus, it has been shown that MRP overexpression or changes in topoisomerase

II activity cannot account for the MDR phenotype observed in the P-gp-negative 2R120 and GLC4/ADR cells, in which the role of an additional mechanism(s) has been proposed (Kuiper *et al*., 1990; Zijlstra *et al*., 1987). TAP seems to be a good candidate. Why TAP appears to be less frequently up-regulated in P-gp-positive MDR cell lines remains unclear. Another possibility for the joing up-regulation of TAP and MRP, and less frequently of TAP and P-gp, is that different ABC transporters share similar transcription machinery (i.e. similar transcription factors or promoter regions). This possibility could be further investigated by measuring mRNA levels and/or rates of transcription in nuclear run-on experiments.

In conclusion, we have shown TAP overexpression in cancer cell lines selected in the laboratory for MDR phenotype, and have provided additional data supporting the capacity of TAP to confer drug resistance. Further insight into the potential contribution of TAP to MDR, and into the functional significance of TAP-MHC I overexpression in MDR cancer cell lines may require the study of other *TAP1*+2 transfectants, the specific suppression of *TAP1/2* gene expression in TAP-overexpressing MDR cells, as well as the study of TAP expression in Pgp- and MRP-negative MDR cancer cell lines (Takeda *et al*., 1994; Slapak *et al*., 1994).

**Acknowledgements**
This work was supported by grants from the Spanish Ministry of Education and Science and from the European Cancer Center, Amsterdam (to MAI). The authors would like to thank HL Ploegh for supplying polyclonal TAP1 serum, and HJ Broxterman, EGE de Vries, MS Center and WS Dalton for providing the different MDR cancer cell lines.

### References

BECK WT AND DANKS MK. (1991). Characteristics of multidrug resistance in human tumor cells. In *Molecular and Cellular Biology of Multidrug Resistance in Tumor Cells*, Roninson IB. (ed.) pp. 3–55. Plenum: New York.

CHILDS S AND LING V. (1994). The MDR superfamily of genes and its biological implications. In *Important Advances in Oncology*, DeVita VT, Hellman S and Rosenberg SA. (eds) pp. 21–36. Lippincott: Philadelphia.

COLE SPC, BHARDWAJ G, GERLACH JH, MACKIE JE, GRANT CE, ALMQUIST KC, STEWART AJ, KURZ EU, DUNCAN AMV AND DEELEY RG. (1992). Overexpression of a transporter gene in a multidrug-resistant human lung cancer cell line. *Science*, **258**, 1650–1654.

COLE SPC, SPARKS KE, FRASER K, LOE DW, GRANT CE, WILSON GM AND DEELEY RG. (1994). Pharmacological characterization of multidrug resistant MRP-transfected human tumor cells. *Cancer Res*., **54**, 5902–5910.

CROMME FV, AIREY J, HEEMELS MT, PLOEGH HL, KEATING PJ, STERN PL, MEIJER CJ AND WALBOOMERS JM. (1994). Loss of transporter protein, encoded by the TAP-1 gene, is highly correlated with loss of HLA expression in cervical carcinomas. *J. Exp. Med*., **179**, 335–340.

EYTAN GD, BORGNIA MJ, REGEV R AND ASSARAF YG. (1994). Transport of polypeptide ionophores into proteoliposomes reconstituted with rat liver P-glycoprotein. *J. Biol. Chem*., **269**, 26058–26065.

FLENS MJ, IZQUIERDO MA, SCHEFFER GL, FRITZ JM, MEIJER CJLM, SCHEPER RJ AND ZAMAN GJ. (1994). Immunochemical detection of MRP in human multidrug-resistant tumor cells by monoclonal antibodies. *Cancer Res*., **54**, 4557–4563.

GOTTESMAN MM AND PASTAN I. (1993). Biochemistry of multidrug resistance mediated by the multidrug transporter. *Annu. Rev. Biochem*., **62**, 385–427.

GRANT CE, VALDIMARSSON G, HIPFNER DR, ALMQUIST KC, COLE SPC AND DEELEY RG. (1994). Overexpression of multidrug resistance-associated protein (MRP) increases resistance to natural product drugs. *Cancer Res*., **54**, 357–361.

GUILD BC, MULLIGAN RC, GROS P AND HOUSMAN DE. (1988). Retroviral transfer of a murine cDNA for multidrug resistance confers pleiotropic drug resistance to cells without prior drug selection. *Proc. Natl Acad. Sci. USA*, **85**, 1595–1599.

HAMADA H AND TSURUO T. Functional role for the 170 to 180 kDa glycoprotein specific to drug resistant tumor cells as revealed by monoclonal antibodies. *Proc. Natl Acad. Sci. USA*, **83**, 7785–7789.

HIGGINS CF. (1992). ABC transporters: from microorganisms to man. *Annu. Rev. Cell Biol*., **8**, 67–113.

KUIPER CM, BROXTERMAN HJ, BAAS F, SCHUURUHUIS GJ, HAISMA HJ, SCHFFER GL, LANKELMA J AND PINEDO HM. (1990). Drug transport variants without P-glycoprotein overexpression from a human squamous lung cancer cell line after selection with doxorubicin. *J. Cell Pharmacol*., **1**, 35–41.

MANAVALAN P, SMITH AE AND MCPHERSON JM. (1993). Sequence and structural homology among membrane-associated domains of CFTR and certain transporter proteins. *J. Prot. Chem*., **12**, 279–290.

MARQUARDT D AND CENTER MS. (1992). Drug transport mechanisms in HL60 cells isolated for resistance to adryamicin: evidence for nuclear drug accumulation and redistribution in resistant cells. *Cancer Res*., **52**, 3157–3163.

MOMBURG F, ORTIZ-NAVARRETE V, NEEFJES J, GOULMY E, VAN DE WAL Y, SPITS H, POWIS SJ, BUTCHER GW, HOWARD JC, WALDEN P AND HAMMERLING J. (1992). Proteasome subunits encoded by the major histocompatibility complex are not essential for antigen presentation. *Nature*, **360**, 174–177.

NEEFJES JJ AND PLOEGH HL. (1988). Allele and locus-specific differences in cell surface expression and the association of HLA class I heavy chain with beta 2-microglobulin: differential effects of inhibition of glycosilation on class I subunit association. *Eur. J. Immunol*., **18**, 801–810.

NEEFJES JJ, MOMBURG F AND HÄMMERLING GJ. (1993). Selective and ATP-dependent translocation of peptides by the MHC-encoded transporter. *Science*, **261**, 769–771.

NEISAG A, ROELFE G, SIJTS AJAM, OSSENDORP F, FEELTKAMP MCW, KAST WM, MELIEF CJM AND NEEFJES JJ. (1995). Major differences in transport-associated with antigen (TAP)-dependent translocation of MHC class I. Presentable peptides and the effect of flanking sequences. *J. Immunol*., **154**, 1273–1279.

RESTIFO NP, ESQUIVEL F, KAWAKAMI Y, YEWDELL JW, MULE JJ, ROSENBERG SA AND BENNINK JR. (1993). Identification of human cancers deficient in antigen processing. *J. Exp. Med*., **177**, 265–272.

SALTER RD, HOWELL DN AND CRESSWELL P. (1985). Genes regulating HLA class I antigen expression in T-B lymphoblast hybrids. *Immunogenetics*, **21**, 235–246.

SARKADI BS, MÜLLER M, HOMOLYZ L, HOLLO Z, SEPRODI J, GERMANN VA, GOTTESMAN MM, PRICE EM AND BAUCHER RC. (1994). Interaction of bioactive hydrophobic peptides with the human multidrug transporter. *FASEB J*., **8**, 766–770.

SCHEPER RJ, BULTE JW, BRAKKEE JG, QUAK JJ, VAN DER SCHOOT E, BALM AJ, MEIJER CJ, BROXTERMAN HJ, KUIPER CM, LANKELMA J AND PINEDO HM. (1988). Monoclonal antibody JSB-1 detects a highly conserved epitope on the P-glycoprotein associated with multidrug-resistance. *Int. J. Cancer*, **42**, 389–394.

SCHUURHUIS GJ, BROXTERMAN HJ, DE LANGE JHM, PINEDO HM, VAN HEIJNINGEN TH, KUIPER CM, SCHEFFER GL, SCHEPER RJ, VAN KALKEN CK, BAAK JP AND LANKELMA J. (1991). Early multidrug resistance, defined by changes in intracellular doxorubicin distribution, independent of P- glycoprotein. *Br. J. Cancer*, **64**, 857–861.

SHARMA RC, INOUE S, ROITELMAN J, SCHIMKE RT AND SIMONI RD. (1992). Peptide transport by the multidrug resistance pump. *J. Biol. Chem*., **267**, 5731–5734.

SLAPAK CA, FRACASSO PM, MARTELL RL, TOPPMEYER DL, LECERF JM AND LEVY SB. (1994). Overexpression of the multidrug resistance-associated protein (MRP) gene in vincristine but not doxorubicin-selected multidrug-resistant murine erythroleukemia cells. *Cancer Res*., **54**, 5607–5613.

STUTTS MJ, GABRIEL SE, OLSEN JC, GATZY JT, O'CONNELL TL, PRICE EM AND BOUCHER RC. (1993). Consequences of heterologous expression of the cystic fibrosis transmembrane conductance regulator in fibroblasts. *J. Biol. Chem.*, **268,** 20653–20658.

TAKEDA Y, NISHIO K, NIITANI H AND SAIJO N. (1994). Reversal of multidrug-resistance by tyrosine–kinase inhibitors in a non-P-glycoprotein-mediated multidrug-resistant cell line. *Int. J. Cancer*, **57,** 229–239.

WEI LY, STUTTS MJ, HOFFMAN MM AND ROEPE PD. (1995). Overexpression of the cystic fibrosis transmembrane conductance regulator in NIH 3T3 cells lowers membrane potential and intracellular pH and confers a multidrug resistance phenotype. *Biophys. J.*, **69,** 883–895.

ZAMAN GJR, FLENS MJ, VAN LEUSDEN MR, DE HAAS M, MULDER HS, LANKELMA J, PINEDO HM, SCHEPER RJ, BAAS F, BROXTERMAN HJ AND BORST P. (1994). The human multidrug resistance-associated protein (MRP) is a plasma membrane drug efflux pump. *Proc. Natl Acad. Sci. USA*, **91,** 8822–8826.

ZIJLSTRA JG, DE VRIES EGE AND MULDER NH. (1987). Multifactorial drug resistance in an adriamycin-resistant human small cell lung carcinoma cell line. *Cancer Res.*, **47,** 1780–1784.